# Psychoanalytic and cognitive-behavior therapy of chronic depression: study protocol for a randomized controlled trial

**DOI:** 10.1186/1745-6215-13-117

**Published:** 2012-07-26

**Authors:** Manfred E Beutel, Marianne Leuzinger-Bohleber, Bernhard Rüger, Ulrich Bahrke, Alexa Negele, Antje Haselbacher, Georg Fiedler, Wolfram Keller, Martin Hautzinger

**Affiliations:** 1 Department of Psychosomatic Medicine and Psychotherapy, University Medical Center of the Johannes Gutenberg University Mainz, Mainz, Germany; 2 Sigmund-Freund-Institute, Goethe-University Frankfurt, University of Kassel, Frankfurt, Germany; 3 Center for Documentation, Statistics, and Data analyses, Ludwig-Maximilians University Munich, Munich, Germany; 4 Department of Psychosomatic Medicine and Psychotherapy, University Clinic Hamburg-Eppendorf, Hamburg, Germany; 5 Clinics in the Theodor-Wenzel-Werk, Department of Psychosomatic Medicine and Psychotherapy, Berlin, Germany; 6 Section Clinical Psychology & Psychotherapy, University Hospital Tübingen, Tübingen, Germany

**Keywords:** Chronic depression, Cognitive behavioral therapy, Psychodynamic psychotherapy

## Abstract

**Background:**

Despite limited effectiveness of short-term psychotherapy for chronic depression, there is a lack of trials of long-term psychotherapy. Our study is the first to determine the effectiveness of controlled long-term psychodynamic and cognitive-behavioral (CBT) treatments and to assess the effects of preferential vs. randomized assessment.

**Methods/design:**

Patients are assigned to treatment according to their preference or randomized (if they have no clear preference). Up to 80 sessions of psychodynamic or psychoanalytically oriented treatments (PAT) or up to 60 sessions of CBT are offered during the first year in the study. After the first year, PAT can be continued according to the ‘naturalistic’ usual method of treating such patients within the system of German health care (normally from 240 up to 300 sessions over two to three years). CBT therapists may extend their treatment up to 80 sessions, but focus mainly maintenance and relapse prevention. We plan to recruit a total of 240 patients (60 per arm). A total of 11 assessments are conducted throughout treatment and up to three years after initiation of treatment. The primary outcome measures are the Quick Inventory of Depressive Symptoms (QIDS, independent clinician rating) and the Beck Depression Inventory (BDI) after the first year.

**Discussion:**

We combine a naturalistic approach with randomized controlled trials(RCTs)to investigate how effectively chronic depression can be treated on an outpatient basis by the two forms of treatment reimbursed in the German healthcare system and we will determine the effects of treatment preference vs. randomization.

**Trial registration:**

http://www.controlled-trials.com/ISRCTN91956346

## Background

Based on a recent review, each year 38.2% of the EU population suffers from a mental disorder. Depression has been identified as the most disabling disease affecting about 17% of the population during their lifetime [1]. According to the WHO, depression will be the second most frequent illness and will carry the highest load of impairment of quality of life worldwide in 2020. Parental chronic depression is also a heavy burden for children [2]. Only 50% of all depressed patients recover within six months and two-thirds within one year, while 6% to 7% are still depressed after 10 to 15 years. There is a high danger of a chronic course of depression, as 30% of all episodes of depression last longer than two years. Half of the patients relapse after their first episode, 70% after the second, and 90% after their third episode [3]. While medication has evolved as the most frequent first-line approach to the treatment of depression, 20% to 30% of patients have not responded to medication; one-third of those responding relapse within one year, and 75% relapse within five years.

In more than 80 randomized controlled trials (RCTs), cognitive behavioral therapy (CBT) has been established as an effective treatment for depression [4-6]. In a recent meta-analysis [7] based on 23 studies totaling 1,365 subjects, this could also be established for short-term psychodynamic psychotherapy (STPP). Compared with other psychotherapies (usually CBT), a small but significant effect size (*d* = −0.30) was found, indicating the superiority of other psychotherapies immediately post-treatment, but no significant differences were found at 3-month (*d* = −0.05) and 12-month (*d* = −0.29) follow-ups.

So far, the evidence for psychotherapy of depression rests on short-term treatments [6-8]. Unlike the results for the treatment of acute depression, psychotherapy, findings of the effects for chronic depression (dysthymia or major depressive episodes) have been limited [8]. In addition, short-term treatments offered in randomized controlled trials lack external validity. Unlike the treatments performed in clinical trials, psychotherapies in clinical practice throughout Europe are of considerably longer duration (CBT up to 60 sessions, psychodynamic treatment up to 80, psychoanalysis up to 240 to 300 sessions). Positive long-term effects of psychoanalysis and psychoanalytic long-term treatments were shown in a representative, multi-perspective and retrospective study [9]. Yet, up until now, there are no controlled studies available comparing psychoanalysis (PAT) and cognitive behavior therapy (CBT) directly [10,11]. However, a few clinical reports [12] comparing psychoanalytic and cognitive-behavioral long-term therapy exist. They demonstrate that even when psychoanalysts and behavior therapists treat similar disorders with similar success rates, the patients differ in many ways.

### Patient preferences

Patient preferences have been identified as one of the three key components of evidence-based medicine, along with the best available research and clinical expertise. They can be defined as the behaviors or attributes of the therapist or therapy that patients value or desire. Regardless of the therapeutic model, engaging patients to become active in psychotherapy is a crucial variable [13]. Patient preferences are particularly important in psychoanalytic long-term therapies with chronically, severely ill patients who have already undergone several unsuccessful therapies [9].

Role preferences involve the behaviors and activities that clients desire themselves and their therapists to engage in while in therapy (for example, preferring the therapist to take an active advice-giving or a listening role, preferring a group or an individual format). Therapist preferences entail characteristics that clients hope their therapists will possess (for example, extended clinical experience, similar ethnic background). Treatment preferences include specific desires for the type of intervention that will be used (for example, a behavioral or a psychodynamic approach, psychotherapy or pharmacotherapy).

A recent meta-analysis [14] of 35 studies found that patients who were matched to their preferred treatment were less likely to drop out (OR = 0.59; *P* < 0.001) and showed greater improvement (*d* = 0.31; *P* < 0.001). Type of preference (role, therapist, treatment) had no specific effect, but type of design was a significant moderator with greatest effects in RCTs. Assignment by chance may or may not meet a patient’s preference. Patients with strong preferences may refuse randomization and therefore threaten the external validity of an RCT.

Therefore, we decided to use a complex controlled design with a patient’s preference arm (assignment of patients according to their treatment preference) and a randomization arm (assignment of patients by chance). Thus, we are able to include patients who refuse randomization. However, to articulate preferences, patients need to know the alternatives. Even patients who have already undergone psychotherapy are often unable to specify what specific form of treatment they had, for example, both PAT and CBT are described as some kind of talking cure. To capture patients’ preferences, we provided all patients with brief descriptions of both treatments offered in this trial before their decisions for preference of randomization.

## Methods

### Study centers

The study is carried out at the following clinical sites: the Sigmund Freud Institute at Frankfurt, the Department of Psychology at the Frankfurt University, the Department of Psychosomatic Medicine and Psychotherapy at University Medicine Mainz, the Department of Psychology at the University of Mainz, the Department of Psychosomatic Medicine and Psychotherapy at the University Clinic Hamburg-Eppendorf, the Department of Psychosomatic Medicine at University Clinic Benjamin Franklin and clinics in the Theodor-Wenzel-Werk Berlin, and the Department of Psychology at the Free University Berlin. These sites are responsible for screening, information, and independent assessment of all patients treated by licensed psychotherapists (PAT or CBT), either in an outpatient unit or in private practice. We have taken care to include different areas of Germany (middle, and north) and institutions, in order to include a broad range of patients and therapists. The Department of Psychology at the University of Tübingen is responsible for CBT training and supervision at all clinical sites. The center for documentation, statistics, and data analyses at the Ludwig-Maximilians University Munich is an independent site that performs data monitoring, randomization, and statistical evaluation. Patients were recruited through outpatient clinics and private practice. In all cases, assessments are conducted by independent, trained, and supervised clinicians, who are blind to the intervention.

### Participants

We include patients between 21 and 60 years of age suffering from chronic depression. Patients have to be depressed for more than one year and currently meet a diagnosis of major depression or dysthymia. In addition, their current depression has to be of a certain severity, meeting a Beck Depression Inventory (BDI - self-report) score above 17 and aclinician-rated Quick Inventory of Depressive Symptoms (QIDS-C) score of more than 10 points. These inclusion criteria and the exclusion criteria are listed in Table 1. Patients can be on antidepressant medication but still meet the inclusion criteria. Patients on medication have to be on a stable dosage for more than four weeks. After information and discussion of the study protocol, the required assessments over three years, and the study interventions, patients have to sign an informed consent sheet before starting baseline assessment and either preferred or randomized treatment.

**Table 1:** Inclusion and exclusion criteria

Inclusion criteria	▪Diagnosis of major depression or dysthymia (based on Structured Clinical Interview - SCID) for at least 12 months
	▪QIDS-C score > 10; BDI 2 >17
	▪Complaints for at least 12 month
	▪Age: 21 to 60 years
	▪Sufficient knowledge of the German language
	▪Informed consent with the study protocol
Exclusion criteria	▪Current or past psychotic symptomatology, schizoaffective, schizophrenic, or bipolar affective disorder
	▪Substance dependence current or during the last three years
	▪Dementia
	▪Borderline, schizotypal and antisocial personality disorder
	▪Acute suicidality
	▪Restriction of intellectual capacity
	▪Serious physical illness that strongly affects the depression or is causal for the depression
	▪Concurrent psychotherapeutic treatment

### Intervention: psychoanalytic therapy

Psychoanalytic psychotherapeutic and psychoanalytic strategies are well developed for severely and chronically depressed patients with comorbid conditions [15-18]. Psychoanalytic authors always consider depression in the context of developmental processes, particularly pathological processes determined by unconscious fantasies and conflicts (a) concerning a differentiated, integrated, and realistic basic feeling of self and identity; (b) concerning the ability to engage in satisfying reciprocal interpersonal relationships; and (c) concerning the ability to unfold one’s own creativity in work, developmental tasks matching the patient’s lifecycle, and satisfying management of everyday life situations. Discovering the unconscious determining factors due to failures in the development (archaic unconscious fantasies stimulated by traumatizations, pathological relationships, burdened life situations, etc.) and working through idiosyncratic unconscious fantasies and conflicts due to developmental deficits and traumatizations in the ‘here and now’ of the therapeutic relationship is seen as indispensable for a long-lasting change of depressive symptoms. Thus, different forms of depression are not understood as clearly distinct entities due to specific genetic or neurobiological factors, but as products of complex interactions between genetic vulnerabilities and experiences in early relationships leading to pathological fantasies, developments, and adaptations. They constitute maladaptive attempts of the individual to cope with severe and lasting disruptions of his normal development.

All psychoanalytic study therapists were trained in the Tavistock manual for treating chronic depressed patients [18] in a workshop. This manual details psychoanalytic techniques to be applied with this group of patients, illustrated by clinical ‘anchor examples’. Pretested in a British trial with chronically depressed patients, it specifies a therapeutic approach, including establishing emotional contact, receptivity and openness, identification of fears, activity, and work in the ‘here and now’ and in transference. A psychodynamic model of the evolvement of chronic depression provides a background of specific interventions. Participating psychoanalysts have had at least three years of clinical practice, participate in regular supervision groups, and record at least 30 therapy sessions, to permit independent control for adherence and competence.

### Intervention: cognitive behavior therapy

Cognitive and behavior therapy for depression was developed by Beck, Rush, Shaw, and Emery [19] and Lewinsohn, Munoz, Youngren, and Zeiss [20]. Their manuals are adapted and integrated in a widely used and well-accepted CBT for depression [21]. Nearly all licensed behavior therapists are familiar with this manual and material, and have received formal training in using the manual. In general, CBT with depressed patients follows five phases within 25 to 45 sessions:

· Phase 1: Development, biographical information, problem analysis, goals, psychoeducation, rationale for treatment, explanation of intervention steps.

· Phase 2: Behavior oriented interventions, activation, increasing pleasant activities, balance of negative and positive activities, situation analysis, structuring day and week.

· Phase 3: Cognitive interventions, thought control, focus on automatic thoughts and alternatives, influence basic assumptions and schemata.

· Phase 4: Skill training, social skills, problem-solving skills, communication skills, role play, stress management, etc.

· Phase 5: Maintenance, prepare for crisis and beginning depression, relapse prevention, transfer into everyday life.

This basic CBT for depression can be extended for chronic depressed patients by intervention elements and strategies (for example, situational analysis, skill training, disciplined self-disclosure) of cognitive behavioral system of psychotherapy [22]. All the study CBT-therapists are well trained and state licensed. They see patients regularly, either in their own private practice or as therapists in cooperating outpatient units. Furthermore, they all participated in an initiating workshop about CBT of chronic depression and were supervised throughout the study. Supervision was offered at each site by experienced senior behavior therapists. In addition, at each site, additional workshops about CBASP or MBCT, or both, were held for the study therapists. Each therapy session is taped and a selection of tapes of each therapy will be rated to control for adherence to cognitive behavior therapy and for therapists’ competence.

### Assessments

The time points of assessment and the measures are presented in Table 2. Assessment is conducted before assignment to treatments, and over a course of three years. Patients are assessed (structured clinical interview, SCID; quick inventory of depressive symptoms, QIDS) by independent and trained clinicians, who are blind to treatment conditions. We took great care to characterize patients by psychoanalytically oriented characteristics of psychic structure and conflict [23-25].

**Table 2:** Planned assessments in the course of the trial (t7 to t10 are options for extension, depending on funding)

**Measure point**	**Patient**	**Therapist**	**Diagnostician**
**t0 intake**	BDI-2, SCL90-R, DAS, DEQ, CTQ, IIP		SKID I/II, QIDS-C, OPD-2, HUS, SRS, SOFAS
t1 (weekly, weeks 1–6)	QIDS-S, HAQ	QIDS-C, HAQ	
t2 (3 mo)	QIDS-S, HAQ	QIDS-C, HAQ	
t3 (6 mo)	QIDS-S, HAQ	QIDS-C, HAQ	
**t4 main assessment (12 mo)**	BDI-2, SCL-90R, DAS, DEQ, IIP, HAQ	QIDS-C, HAQ	LIFE, QIDS-C, OPD-2, HUS, SRS, SOFAS
t5 (18 mo)	QIDS-S, HAQ	QIDS-C, HAQ	
**t6 main assessment (24 mo)**	BDI-2, SCL-90R, DAS, DEQ, IIP, HAQ		LIFE, QIDS-C, HUS, SRS, SOFAS
t7 (30 mo)	QIDS-S, HAQ	QIDS-C, HAQ	
**t8 main assessment (36 mo)**	BDI-2, SCL-90R, DAS, DEQ, IIP, HAQ	QIDS-C, HAQ	LIFE, QIDS-C, OPD-2, HUS, SRS, SOFAS
**t9 main assessment (48 mo)**	BDI-2, SCL-90R, DAS, DEQ, IIP, HAQ		LIFE, QIDS-C, HUS, SRS, SOFAS
**t10 final assessment (60 mo)**	BDI-2, SCL-90R, DAS, DEQ, IIP, HAQ		LIFE, QIDS-C, OPD-2, HUS, SRS, SOFAS

Notes: *BDI 2* Beck Depression Inventory II, *CTQ* Childhood Trauma Questionnaire, *DAS* Dysfunctional Attitude Scale, *DEQ* Depressive Experience Questionnaire, *HAQ* Helping Alliance Questionnaire (patient-, therapist-form), *HUS* Heidelberger Restructuring Scale, *IIP* Inventory of Interpersonal Problems, *LIFE* Longitudinal Follow-up Evaluation, *OPD-2* Operationalized Psychodynamic Diagnostics, *PEI* Psychoanalytic Intake Interview, *QIDS* Quick Inventory of Depressive Symptoms [self- (QIDS-S) and clinician-rating (QIDS-C)], *SCID I, II* Structured Clinical Interview according to DSM-IV, *SCL-90R* Symptom-Checklist, *SOFAS* Social functioning, *SRS* Self-Reflective Functioning Scale.

### Objectives and hypotheses

The purpose of the trial is to address the following issues:

▪ Short- and long-term efficacy of the two treatments (PAT, CBT).

▪ Course and stability of treatment effects.

▪ Impact of randomization compared with preference for both treatments.

▪ Health costs (work loss, hospitalization, outpatient treatment, drugs, etc.).

### Hypotheses

A. Effectiveness and comparison of the two treatments with the chronically depressed: Both psychotherapies lead to positive effects concerning (a) the reduction of depressive symptoms (QIDS, BDI), (b) the proportion of remitted patients (LIFE), (c) improved social functioning (SOFAS), (d) a decrease of continuous antidepressant medication. These effects are manifested at the main assessments (see Table 2: t4, t6, t8) a cross the trial. The courses of change differ between PAT and CBT: (a) CBT achieves faster symptom reduction, (b) PAT starts slowly but achieves more stable, lasting effects (this is evidenced in the process measures, the rates of response and the symptom level at follow-up (see Table 2: t6, t8, t10).

B. Comparison of preferred and randomized therapies: Preferred therapies are expected to lead to stronger effects and less study drop-outs than randomized therapies. Patient preference is particularly important in PAT. Therefore, we expect larger effect differences between PAT preference and PAT randomization compared with CBT.

C. Health costs and cost effectiveness. We expect a significant reduction of the days of absence from work and of further treatment needed (for example, hospitalization) for both therapies. We expect larger health-cost reductions for PAT compared with CBT in the long term. We expect that treatment costs for CBT will be lower than those for PAT, resulting in a better cost-benefit ratio.

### Design

**Figure 1:**
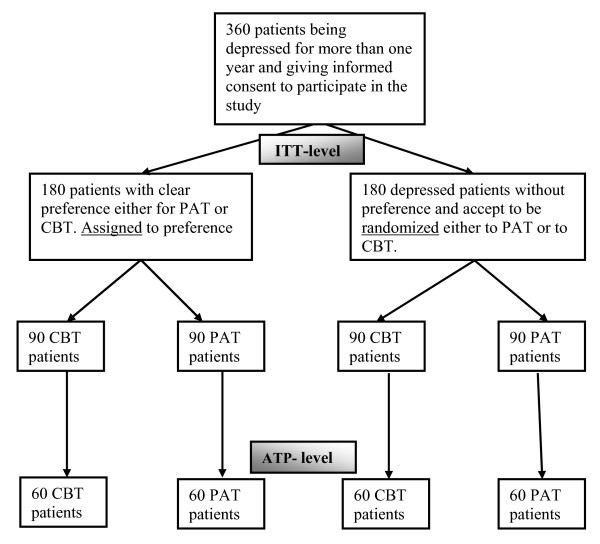
Study design (projected numbers).

The trial will include about 360 chronically depressed outpatients. About 180 patients will have a clear preference foreither PAT or CBT. After baseline assessment (t0) they will be assigned to a study therapist offering the preferred intervention. About 180 patients will come without preference and accept to be randomized to either PAT or CBT. Randomization will take place after baseline assessment (t0). Figure1 summarizes the 2 (preference) by 2 (psychotherapies) by 4 (time) factorial design.

### Outcome

The primary outcome measures are the Quick Inventory of Depressive Symptoms (QIDS [26]) (independent clinician rating) and the Beck Depression Inventory (BDI-2) one year after initiation of treatment (t4). Treatment response is defined as a decline of at least 50% from t0 (baseline, pre-treatment) to t4 (12 months after intake. Remission is defined by a QIDS-score below 6. A relapse is defined when the criteria of a major depressive disorder (MDD) are fulfilled, for example, a QIDS-score above 9 is documented or hospitalization or outpatient treatment are needed.

Secondary outcome measures include the Beck Depression Inventory (BDI 2 [27]), the QIDS-self-rating, the Symptom-Checklist (SCL-90R [28]), the Depressive Experience Questionnaire (DEQ [29]) and the Dysfunctional Attitude Scale (DAS [30]). Furthermore, we assess social functioning (SOFAS [31]), the Helping Alliance Questionnaire (HAQ [32], and recurrence of depressive episodes assessed by the longitudinal follow-up evaluation interview (LIFE [33]).

### Sample size calculation

Sample size calculation was based on meta-analyses with similar interventions in similar patient groups using similar outcome instruments [10,11,34,35]. Based on the Hamilton Depression Rating Scale (a 17-item clinician rating form, similar to the QIDS-C) we expect pre-post effect sizes for psychotherapies of depression of *d* = 1.2. A study including a direct comparison of PAT and CBT is not available. Therefore, we used meta-analysis of comparing psychotherapy with psychiatric therapy with depressed out-patients to extrapolate a probable effect size of *d* = 0.50 between treatments of different efficacy at post-treatment. To detect this difference at *α* = 0.025 (two-tailed) with a power of 0.80, a minimum number of 60 patients per cell are required (see Figure1).

### Secondary analyses

Self-report questionnaires and observer ratings will be analyzed by mixed models with repeated measurements. With two co-primary outcomes, we will conduct the tests at a 0.025 level of significance (two-tailed) to adjust for the multiple comparison issue. Further analyses will include cross-sectional analyses to compare therapists; subgroup analyses of subjects’ and therapists’ conditions; and costs of the SP treatment.

Descriptive statistics showing the measurements over time will be presented whenever appropriate. Serious adverse events and cases of drop-outs will be analyzed descriptively.

### Randomization

As Figure1 shows, in a partial randomization preference trial, patients are asked if they have a preference for one specific treatment (PAT or CBT). Treatments are outlined to them in terms of a general description (see box for the instructions). If they articulate a specific preference, they are assigned accordingly (preference arm). If they articulate no specific preference, they are randomized (randomization arm).

### Statistical analysis

The design is an analysis of variance design with repeated measures: The main factor is the form of treatment (CBT or PAT) and assignment (preference or randomization). Four main assessments encompass the treatment phase and follow-up. The variability of intake symptoms is controlled as a covariate. To determine the time factor, ANCOVAs with repeated measures and linear regression with autocorrelated data (compound symmetric covariance structure models) are applied. Dichotomous response scores are examined by tests for frequency distributions and procedures of survival analysis (Coxregression, log-ranktests). It is also tested whether differences between CBT and PAT depend on assignment (preference vs. randomization), that is, if a naturalistic selection brings clearer differences than random assignment.

### Safety aspects

Safety parameters will comprise newly occurring psychiatric diagnoses (LIFE) and all serious adverse events that are reported during and up to six months after treatment.

### Medical complications

The recording of adverse events will be restricted to psychological conditions. Formally, these are defined as any disorder classified by the International Classification of Diseases F00 to F99. A serious adverse eventis an adverse event that may occur at any time of the treatment phase or up to six months after the end of treatment, that: results in death; is life-threatening; requires subject hospitalization or prolongation of existing hospitalization; results in persistent or significant disability or incapacity; or is a congenital anomaly or birth defect.

Any adverse event (according to the study specific definition) reported by the subject or detected by the local investigator will be collected during the trial and must be documented in the CRF. The local investigator will use ICD-10 to code the event. The clinical course of the adverse event will be followed until it has changed to a stable condition or until the end of follow-up phase, whichever comes first. In case of serious adverse events, the Ethics Committee and Data Monitoring Safety Board (DMSB) will be informed within 24 hours of the serious adverse event becoming known.

### Ethical issues

The final study protocol and the final version of the written informed consent form were approved by the Ethics Committee of the Federal State of Rhineland Palatine in Germany (ref: 837.124.075659). The procedure set out in this protocol, pertaining to the conduct, evaluation, and documentation of this trial, were designed to ensure that all persons involved in the trial abide by good clinical practice and the ethical principles described in the current revision of the Declaration of Helsinki. The trial will be carried out in keeping with local legal and regulatory requirements. Before being admitted to the clinical trial, patients must consent to participate after the nature, scope, and possible consequences of the clinical trial have been explained in a form understandable to them. The patients must give written informed consent to participate in the study, including their consent to publish.

## Discussion

This trial has the major goals: (1) to determine the effectiveness of the two long-term treatments (PAT, CBT) reimbursed in the German healthcare system for chronic depression, and (2) to compare the effects of treatment preference against randomization. It combines the virtues of a randomized controlled (efficacy) trial and a naturalistic approach.

As recent meta-analyses have shown, the treatment of depression by short-term treatments has been plagued by high relapse rates and a lack of lasting effectiveness. Long-term PAT and CBT, however, are not easy to compare, as they differ in frequency (one to three sessions per week), total number of sessions, and duration of treatment (one to three years). We therefore tried to reach comparable doses in the first year of treatment. Initially, psychoanalysts often work with low frequency (≤2 sessions per week) with depressed patients; thus there could be 45 sessions of CBT and up to 80 sessions of PAT in the first year. As our primary outcome is measured at one year (t4), doses of treatment can be compared. When psychoanalytic treatment is continued after one year, dose can be adjusted according to patient need and treatment process. As CBT entails relapse prevention, there will also be an extended treatment duration. In any case, number and timing of sessions will be documented. In this trial, a high quality is assured by an independent assessment.

While patient preferences form one of the three key components of evidence-based medicine, studies on the impact of patient preference on outcome have been scarce. Our design enables us to determine the impact of patient preference on outcome, and we expect a lower attrition rate and a higher effectiveness for preferred vs. randomized assignment.

As this trial has been funded substantially by the German Society for Psychoanalysis, Psychotherapy, Psychosomatics, and Depth Psychology (DGPT), a great effort was made to counteract allegiance effects. The study also is financially supported by the independent, medically oriented Heidehof Foundation (part of the well known Robert Bosch Foundation, Stuttgart). One of the principal investigators (MH) is a renowned cognitive behavior therapist. Data collection, monitoring, and analysis are performed by an independent statistician (BR).

## Trial status

Ongoing recruitment.

## Abbreviations

ANCOVA, analysis of covariance; BDI 2, Beck depression inventory II; CBT, Cognitive behavioral therapy; CTQ, Childhood trauma questionnaire; DAS, Dysfunctional attitude scale; DEQ, Depressive Experience Questionnaire; DMSB, Data management and safety board; EU, European Union; HAQ, Helping alliance questionnaire (patient-, therapist-form); HUS, Heidelberger restructuring scale; ICD-10, International classification of diseases, 10th revision; IIP, Inventory of interpersonal problems; LIFE, Longitudinal follow-up evaluation; MDD, Major depressive disorder; OPD-2, Operationalized psychodynamic diagnostics; PAT, Psychoanalytically oriented treatments; PEI, Psychoanalytic Intake Interview; QIDS-C, Quick inventory of depressive symptoms, clinician rating; QIDS-S, Quick inventory of depressive symptoms, self-report; RCT, Randomized controlled trials; SCID I, II, Structured clinical interview according to DSM-IV; SCL-90R, Symptom-checklist; SOFAS, Social functioning; STPP, Short-term psychodynamic psychotherapy; SRS, Self-reflective functioning scale.

## Competing interests

The authors declare that they have no competing interests.

## Authors’ contributions

The study design was developed by MEB, ML-B and MH (CBT rationale and procedure), WK (project leaders), and BR (statisticians). Assessments were conceptualized and developed by AH, UB, and AN. MEB and MH wrote an outline of the paper, which was carefully revised, edited, and discussed by MLB, UB and AN (psychoanalytic treatment), BR (statistical rationale and presentation), AH, GF, and WK. All authors read and approved the final manuscript.
